# Significance of EGFRvIII Presence in Gastric Adenocarcinoma

**DOI:** 10.1155/grp/5870339

**Published:** 2026-02-23

**Authors:** Ali Zare-Mirzaie, Narjes Safari, Amirhosein Mehrtash, Nasrin Shayanfar, Ensieh Jafari, Elmira Gheytanchi

**Affiliations:** ^1^ Department of Pathology, School of Medicine, Iran University of Medical Sciences, Tehran, Iran, iums.ac.ir; ^2^ Oncopathology Research Center, Iran University of Medical Sciences, Tehran, Iran, iums.ac.ir; ^3^ Department of Molecular Medicine, Biotechnology Research Center, Pasteur Institute of Iran, Tehran, Iran, pasteur.ac.ir; ^4^ Department of Pathology Rasoul-e-Akram Hospital, Iran University of Medical Sciences, Tehran, Iran, iums.ac.ir

**Keywords:** EGFRvIII variant, epidermal growth factor receptor (EGFR) gene, gastric adenocarcinoma

## Abstract

**Background:**

The significance of epidermal growth factor receptor (EGFR) gene variants, particularly the variant known as EGFRvIII, which arises from the deletion of exons 2–8, has been extensively studied in various human carcinomas. In contrast, studies into the role of EGFRvIII in gastric cancer remain limited. The current study aims to evaluate the expression levels of EGFRvIII in patients suffering from gastric adenocarcinoma.

**Material and methods:**

In this descriptive cross‐sectional study, 21 patients with gastric adenocarcinoma who referred to the university‐based referral hospital, Rasoul‐Akram, in Tehran, Iran, during 2018–2019, were included. The patients’ characteristics including demographic features, medical archives, type and location of tumor mass, and histopathological type were recorded. In order to evaluate the expression of EGFRvIII variant, RT‐PCR and sequencing techniques were used.

**Results:**

In patients with gastric adenocarcinoma, 14 cases (66.7%) were males and seven cases (33.3%) were females, and the average age of the patients was 66.62 ± 10.79 years, ranging from 45 to 89 years. In terms of the histopathological type, six cases (28.6%) were moderately differentiated (MD), four cases (19.0%) were poorly differentiated (PD), five cases (23.8%) were SIG adenocarcinoma, and six cases (28.6%) were well differentiated (WD). A total of 18 samples (85.7%) were obtained from the biopsy, while three cases (14.3%) were derived from total gastrectomy and necrosis was seen in eight cases (38.1%). Two out of 21 cases (9.5%) were EGFRvIII positive in both RT‐PCR and sequencing test. The first case was an 86‐year‐old woman with a signet‐ring cell (SIG) adenocarcinoma tumor subtype with tissue from the antrum and without necrosis. The second case was a 67‐year‐old man with a tumor subtype of SIG adenocarcinoma with necrotic tissue sample from the gastric cavity. The location of the mutation, two genes on ex1 and ex8, which were connected to each other, was detected in samples of both cases.

**Conclusion:**

Our findings showed detection of EGFRvIII in 9.5% of gastric adenocarcinoma cases. Although the sample size presents a limitation, the increase in expression of this variant appears to be independent of the patients’ gender and age. Notably, the association of this variant with the signet‐ring cell adenocarcinoma subtype is a significant finding that warrants further investigation.

## 1. Introduction

Gastric cancer (GC) ranks as the fifth most prevalent malignancy globally and is the third leading cause of cancer‐related mortality. Factors contributing to the risk include *Helicobacter pylori* infection, age, high salt intake, and a diet low in fruits and vegetables [1, 2].

Despite often being asymptomatic in early stages, GC carries a high mortality due to late diagnosis, with over 784,000 deaths reported worldwide [2, 3]. More than 90% of gastric tumors are malignant, of which 95% are malignant adenocarcinoma cases [4]. Various histopathological classification frameworks for GC have been developed. The World Health Organization (WHO) and the Japanese Gastric Cancer Association classifications [5] are the most widely utilized, exhibiting considerable overlap. These classifications categorize GC into three fundamental subtypes: intestinal, diffuse, and mixed. The WHO classification, which is predominantly applied in Western countries [6], identifies five key histological subtypes: tubular, papillary, poorly cohesive (including SIG [SIG] and other forms), mucinous, and mixed adenocarcinomas [7]. The molecular profiles of GC that have been recently identified are essential for a deeper understanding of its subtypes [8]. They may also prove beneficial in pinpointing clinically relevant biomarkers and new therapeutic targets [8]. Moreover, the heterogeneity found both within individual tumors and among different tumors in gastric carcinoma creates significant obstacles in diagnosis and therapy [8].

Four distinct molecular subtypes have been identified by the Cancer Genome Atlas (TCGA) research network including gnomically stable (GS), microsatellite instability‐high (MSI‐H), EBV positive, and tumors exhibiting chromosomal instability (CIN), which are associated with specific molecular abnormalities as well as some overlap among them [2]. The CIN subtype is particularly enriched in copy number variations affecting significant receptor tyrosine kinase oncogenes, such as epidermal growth factor receptor (EGFR), fibroblast growth factor receptor 2 (FGFR2), human epidermal growth factor receptor 2 (HER2), and MET [2]. The EGFR receptor is an oncogene that consists of 28 exons with a length of Kb188 and is located on chromosome 7 as a significant component of the ErbB proto‐oncogene family, which is implicated in the pathophysiology of various epithelial‐origin malignancies [9]. Numerous studies have established a correlation between EGFR and patients’ unfavorable outcomes [10–13]. Furthermore, detecting abnormalities in EGFR gene may help in managing the cancer, particularly in case of targeted therapies. The most prevalent variant of the second extracellular EGFR is EGFRvIII, which is produced by the modification of exons 2–7 in the wild‐type EGFR [14]. EGFR as a receptor tyrosine kinase plays a vital role in the development and maintenance of epithelial and epidermal tissues [15]. Mutations and detection of EGFR are identified as significant contributors in the progression of various cancers, including colorectal cancer (CRC) [16, 17], non‐small cell lung cancer (NSCLC) [18–21], breast cancer [22], head and neck squamous cell carcinomas (HNSCC) [23], and glioblastomas [24], with more than 90% within the intracellular kinase domain. However, more comprehensive investigations, including those utilizing the RT‐PCR method, have revealed that the expression EGFRvIII variant in breast cancer is approximately 68% [17]. The expression of the EGFRvIII variant has been investigated in relation to targeted treatments involving anti‐EGFR drugs (Cetuximab) specifically the extracellular domain, which is applied in management of squamous cell carcinomas in the head and neck region [25]. In light of the high incidence and mortality rates of gastric adenocarcinoma (GA) in Iran, as well as the critical role of the EGFRvIII variant in patient survival and treatment efficacy, this study was designed to evaluate the prevalence of the EGFRvIII in Iranian patients with GA. A notable aspect of this study was its methodological approach; we employed PCR techniques and sequencing to assess the EGFRvIII, in contrast to other previous studies that primarily relied on immunohistochemistry (IHC), as the foundational method.

## 2. Materials and Methods

### 2.1. Patients’ Characteristics and Sample Selection

In this descriptive cross‐sectional study, patients with GA who referred to the university‐based referral hospital, Rasoul‐Akram, in Tehran, Iran, during 2018–2019, were included. The patients’ characteristics including demographic features, medical archives, type and location of tumor mass, and histopathological type were recorded. The study cohort was limited due to strict inclusion criteria, including availability of high‐quality RNA and patient consent, as well as the rarity of certain histologic subtypes within the study period.

### 2.2. Quantitative Real‐Time PCR Analysis and Sequencing

The presence of the EGFRvIII variant was evaluated by RT‐PCR and sequencing techniques. To perform the real‐time qRT‐PCR technique, RNA samples were first extracted from the tumor tissues (30–40 mg in weight and 4–6 mm in thickness) using Qiagen kit. Then, the cDNA was synthetized from RNA using the Quanti‐Tect Reverse Transcription Kit. The PCR product was prepared from cDNA using SYBR Select Master Mix and selected primers.

F‐EXON 1: 5 ^′^‐GAGTCGGGCTCTGGAGGAAA‐3 ^′^

R‐EXON 8: 5 ^′^‐CCATCTCATAGCTGTCGGGCC‐3 ^′^

R‐EXON2: 5 ^′^‐CAGTTATTGAACATCCTCTGGAG‐3 ^′^

To perform the real‐time polymerase chain reaction (qRT‐PCR), the total volume of 50 *μ*L master mix solution was prepared using the amounts of 25 *μ*L master mix, 25 mM MgCl2, and 10 *μ*M of each forward (1 *μ*L) and reverse (1 *μ*L) primers and were then subjected to 25 and 35 amplification cycles of 95°C for 20–30 s and then 95°C for 15 min (Denaturation phase), 50°C–65°C for 20–40 s (Annealing phase), 72°C for 30–90 s (Extension phase), and 72°C for 5 min (Final elongation phase) on the Rotor‐Gene Q Light Cycler (Qiagen, Germany). Finally, to confirm the result of RT‐PCR, the samples were sent for sequencing and the location of the mutation, two genes on ex1 and ex8, which were connected to each other, were detected in this evaluation. In addition to gastric carcinoma specimens, glioblastoma multiforme (GBM) tissue previously confirmed to harbor the EGFRvIII variant was included as an independent positive control to validate the specificity of the assay. No‐template controls (NTCs) were also run in parallel to exclude contamination and nonspecific amplification. In future studies, we plan to incorporate a broader panel of independent EGFRvIII‐positive and ‐negative tissue and cell line controls to further strengthen assay validation and reproducibility.

### 2.3. Statistical Analysis

Statistical evaluation of all data was conducted using SPSS software version 22.0 (SPSS, Inc., IBM Corp, USA). Because of the small number of EGFRvIII‐positive cases, all statistical tests are exploratory and *p* values are reported as descriptive rather than confirmatory inferential statistics. The qualitative data were characterized by frequency (%) and the quantitative data were analyzed using the mean and standard deviation for normally distributed parameters, or the median and the first and third quartiles for those that were not normally distributed. A *p* value of less than 0.05 was deemed significant.

## 3. Results

### 3.1. Patients’ Clinicopathological and Tumor Characteristics

**Figure 1:**
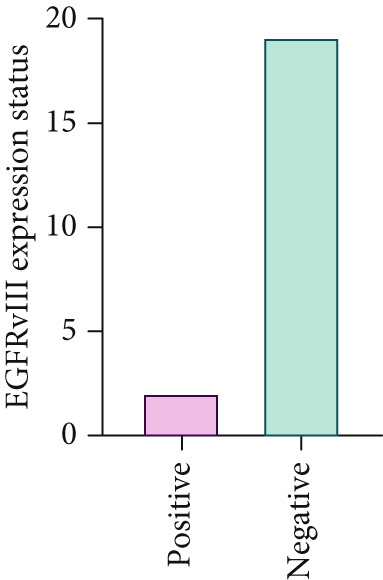
Frequency of patients with gastric adenocarcinoma and EGFRvIII expression.

A total of 21 tumor samples were analyzed for EGFRvIII expression, two out of 21 cases (9.5%) were EGFRvIII positive (both based on RT‐PCR and sequencing). The first case was an 86‐year‐old woman with a SC adenocarcinoma tumor subtype with tissue sample that was taken from the antrum without necrosis, and the second case was a 67‐year‐old man with a tumor subtype of SIG adenocarcinoma with necrotic sample from the gastric cavity, Figure 1.

In patients with GA, the mean age of the patients was 66.62 ± 10.79 years, ranging from 45 to 89 years. Among patients aged ≤ mean age, nine (42.9%) were EGFRvIII positive and 12 (57.1%) were negative, whereas among those > mean age, 0 (0.0%) were positive and two (100.0%) were negative. This difference was not statistically significant (*p* = 0.47, Fisher’s exact test). Regarding gender, 14 (66.7%) of the samples were from males and seven (33.3%) from females. EGFRvIII positivity was observed in 14 males (66.7%) and seven females (33.3%), with no significant difference between genders (*p* = 1.00, Fisher’s exact test).

Most samples were obtained via biopsy (18, 85.7%), with only three (14.3%) from total gastrectomy specimens. EGFRvIII positivity did not differ significantly by sample type (*p* = 1.00, Fisher’s exact test). Tumor location was distributed as follows: cardia (4, 19.0%), body (9, 42.9%), antrum (7, 33.3%), and pylorus (1, 4.8%). EGFRvIII expression was highest in tumors of the cardia (4/4, 21.1%), followed by the body (8/9, 42.1%), antrum (6/7, 31.6%), and pylorus (1/1, 5.3%), with no statistically significant association between location and EGFRvIII positivity (*p* = 0.86, Chi‐square test).

Regarding histological subtype, moderately differentiated (MD) and well‐differentiated (WD) tumors each accounted for six samples (28.6%), poorly differentiated (PD) and SIG tumors accounted for four samples each (19.0%), and one tumor was of the mucinous/signet type (SIG, 4.8%). EGFRvIII positivity tended to be more frequent in WD and SIG tumors, but this trend did not reach statistical significance (*p* = 0.05, Chi‐square test). Tumor necrosis was present in eight samples (38.1%) and absent in 13 (61.9%). EGFRvIII expression was observed in seven necrotic tumors (36.8%) and 12 non‐necrotic tumors (63.2%), with no significant association between necrosis and EGFRvIII status (*p* = 1.00, Fisher’s exact test).

**Figure 2:**
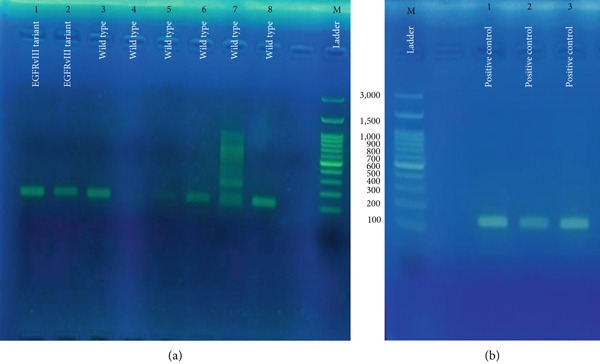
Detection of EGFRvIII expression in gastric carcinoma tissues by RT‐PCR. Primers were designed on exon 1, exon 2, and exon 8 of the EGFR gene. Because of RNA fragmentation in FFPE tissues, the wild‐type allele produced a shorter fragment (111 bp), while the EGFRvIII variant generated a longer fragment (160 bp). (a) Lanes 1–2 show mutant, confirming EGFRvIII expression and Lanes 3–8 wild‐type gastric carcinoma samples, and (b) positive controls (Lanes 1–3) of gastric carcinoma and glioblastoma multiforme (GBM).

Overall, EGFRvIII expression was not significantly associated with patient age, gender, sample type, tumor location, histological subtype, or necrosis. The clinicopathological features of patients are summarized in Table 1. The location of the mutation, two genes on ex1 and ex8, which were connected to each other, was detected in both cases, Figure 2. Given the pilot design and limited sample size, these findings should be interpreted cautiously within the exploratory framework of this study.

**Table 1:** The clinicopathological features of patients with gastric cancer according to EGFRvIII expression.

**Tumor characteristics**	**Total samples** **N** **(%)**	**EGFRvIII negative** **N** **(%)**	**EGFRvIII positive** **N** **(%)**	**p** **value (test)**
Mean age (±SD), years (range)	66.62 ± 10.79			0.47 (Fisher)
≤ Mean age	9 (42.9) 12	9 (47.4)	0 (0.0)	
> Mean age	(57.1)	10 (52.6)	2 (100.0)	
Gender				1.00 (Fisher)
Male	14 (66.7)	13 (68.4)	1 (50.0)	
Female	7 (33.3)	6 (31.6)	1 (50.0)	
Sample type				1.00 (Fisher)
Biopsy	18 (85.7)	16 (84.2)	2 (100.0)	
Total gastrectomy	3 (14.3)	3 (15.8)	0 (0.0)	
Tumor location				0.86 (Chi^2^)
Cardia	4 (19.0)	4 (21.1)	0 (0.0)	
Body	9 (42.9)	8 (42.1)	1 (50.0)	
Antrum	7 (33.3)	6 (31.6)	1 (50.0)	
Pylorus	1 (4.8)	1 (5.3)	0 (0.0)	
Tumor subtype				0.05 (Chi^2^)
MD	6 (28.6)	6 (31.6)	0 (0.0)	
WD	6 (28.6)	4 (21.1)	2 (100.0)	
PD	4 (19.0)	4 (21.1)	0 (0.0)	
SC	4 (19.0)	2 (10.5)	2 (100.0)	
SIG	1 (4.8)	1 (5.3)	0 (0.0)	
Tumor necrosis				1.00 (Fisher)
Present	8 (38.1)	7 (36.8)	1 (50.0)	
Absent	13 (61.9)	12 (63.2)	1 (50.0)	

*Note:* Values are given as number (percentage). Statistical tests: Fisher’s exact test used for 2 × 2 tables; Chi‐square test for variables with more than two categories.

Abbreviations: MD = moderately differentiated, PD = poorly differentiated, SC = signet‐cell carcinoma, SIG = signet‐ring cell, WD = well differentiated.

### 3.2. Evaluation of EGFRvIII Expression Profiles in Gastric Carcinoma Tissue Specimens

**Figure 3:**
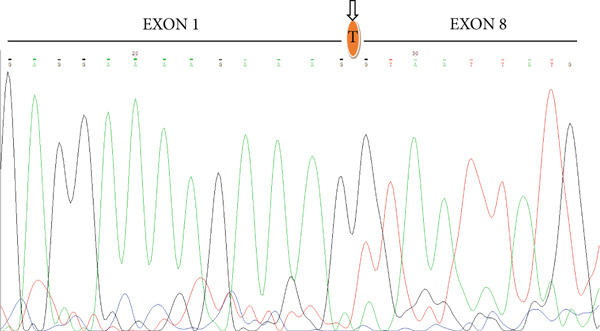
Validation of EGFRvIII transcript by Sanger sequencing. A representative electropherogram demonstrating the EGFRvIII junction between exon 1 and exon 8 is shown. The arrow mark indicates the breakpoint corresponding to the exons 2–7 deletion (EGFRvIII).

The expression of EGFRvIII in gastric carcinoma tissues was assessed by RT‐PCR followed by gel electrophoresis, as illustrated in Figure 2. Specific primers were designed on exon 1, exon 2, and exon 8 of the EGFR gene. Given RNA fragmentation in FFPE tissues, the wild‐type allele yielded a shorter amplicon of 111 bp, whereas the EGFRvIII variant generated a longer fragment of 160 bp. In Figure 2a, Lanes 1 and 2 correspond to samples positive for the EGFRvIII variant, confirming successful amplification of the 160 bp fragment. Lanes 3–8 represent gastric carcinoma specimens that predominantly displayed the wild‐type allele (111 bp). Notably, the variant band was not detected in these samples, suggesting absence of EGFRvIII expression in this subset. Lane M corresponds to the molecular size ladder. In Figure 2b, Lanes 1–3 represent positive control samples, including gastric carcinoma and GBM. These controls consistently displayed the expected EGFRvIII variant band at 160 bp, validating the specificity and reliability of the assay. Collectively, these results demonstrate that while EGFRvIII expression was confirmed in a subset of gastric carcinoma samples (Lanes 1–2), the majority of specimens expressed only the wild‐type allele. The positive control panel further corroborates the accuracy of our molecular detection strategy. In the RT‐PCR analysis of EGFRvIII, amplification with primers targeting exon 1 (F‐EXON1: 5 ^′^‐GAGTCGGGCTCTGGAGGAAA‐3 ^′^) and exon 8 (R‐EXON8: 5 ^′^‐CCATCTCATAGCTGTCGGGCC‐3 ^′^) yielded a product of the expected size in a subset of gastric carcinoma samples. To confirm the identity of this product, selected amplicons were subjected to Sanger sequencing. As shown in Figure 3, the sequencing electropherogram clearly demonstrates the junction between exon 1 and exon 8, consistent with deletion of exons 2–7 and the presence of the EGFRvIII variant. A previously characterized GBM specimen harboring EGFRvIII served as an independent positive control, while no amplification was detected in NTCs, supporting the specificity of the assay.

## 4. Discussion

The significant differences in the rates of incidence and prevalence of GA among different communities highlight the impact of geographic and racial attributes on the disease’s occurrence and its outcomes. In this context, it has been established that genomic variations, including the presence of polymorphisms, repetitive sequences, and the deletion of exons in certain genes, occurred in patients with GA. These genetic alterations may serve as potential prognostic factors. The role of EGFR gene variants has been widely investigated in human carcinomas.

The amplification of EGFR in patients with glioblastoma and the deletion of gene exons and the creation of other genomic forms such as EGFRvI (deletion of the N‐terminal part), EGFRVII (deletion of exons 14 and 15), EGFRvIII (deletion of exons 2–7), EGFRvIV (deletion of exons 27–27), and EGFRvV (deletion of exons 25–28) have been identified [22, 25]. The EGFRvIII variant represents the most prevalent alteration in the EGFR gene and serves as the main point of this study. The mechanism by which the EGFRvIII variant effects malignant tumors has been established, indicating that EGFRvIII functions as an oncogene that promotes the proliferation of cancer cells while simultaneously inhibiting their apoptosis. This phenomenon has been extensively documented across various types of malignant tumors; however, there is a limited investigation into the specific role of EGFRvIII in GC [14, 15, 18, 19, 26]. This study presents a significant advantage by using a different methodological framework (PCR together with sequencing) compared with similar studies that have largely relied on IHC for EGFRvIII expression. Our study demonstrated that the expression rate of EGFRvIII in GA samples was 9.5%. Because of the limited size of samples, it is not possible to perform the analytical examinations to evaluate the association between EGFRvIII expression and demographic or clinicopathological characteristics. Our findings showed that EGFRvIII expression was not affected by the patients’ age or gender. In addition, EGFRvIII was detected in males and females of different ages, but the interesting point was that both samples were positive for the SIG cell adenocarcinoma subtype and its role as a prognostic factor can be emphasized, which need more extensive studies on a larger population. Importantly, our study introduces a dual molecular detection approach combining PCR and sequencing, which may complement traditional IHC in assessing EGFRvIII expression. Furthermore, the exclusive detection of EGFRvIII in the SIG subtype of GA underscores a potentially novel molecular signature that may distinguish this histological variant. Although observed in a limited number of cases, EGFRvIII positivity in this subtype may reflect activation of canonical EGFR‐driven oncogenic pathways such as PI3K/AKT and RAS/RAF/MEK/ERK, both of which are well‐established mediators of tumor proliferation, survival, and therapy resistance [27, 28]. In other malignancies, EGFRvIII has been associated with aggressive behavior and poor therapeutic response, prompting interest in EGFRvIII‐directed interventions such as peptide vaccines (e.g., rindopepimut) and emerging immunotherapies [14, 29]. Therefore, if validated in larger cohorts, our findings could pave the way for subtype‐specific prognostic stratification and translational efforts aiming to incorporate EGFRvIII testing and targeted modalities in the management of GA. This observation, together with the consideration of regional variability in sample origin, emphasizes the novelty of our study and its contribution to understanding subtype‐specific oncogenic pathways in GC. Consistently, we also demonstrated 40% expression of EGFRvIII in the positive control samples (glioblastoma), which underscores its prognostic significance [30]. Nevertheless, studies on EGFRvIII expression in adenocarcinoma subtypes remain limited, and available reports indicate a broad spectrum of expression levels across tumor types.

The increase in EGFRvIII expression in GA was reported as 4%, which was almost half of our study, but they showed that EGFR expression had a direct relationship with recurrence‐free survival and overall survival of patients [31]. The expression level of EGFRvIII in colon adenocarcinoma was 34%, which was higher than in GA, but its expression had no association with the survival rate of patients [32]. No positive EGFRvIII expression was confirmed in patients with metastatic CRC [17]. EGFRvIII variant mutation was found in eight of 252 cases (3.2%) of patients with lung cancer [33]. The expression of EGFRvIII variant was observed in 64% of glioblastoma cases, which was much higher than our findings [14]. In the case of breast cancer, the frequency of this variant was 68% and in thyroid papillary, it was 75% [14]. Consequently, the expression levels of EGFRvIII across different carcinoma types exhibit significant variability, and it may serve as a prominent prognostic factor. Our study indicated that the expression of EGFRvIII significantly reduced within the digestive system. However, this observation should not diminish the potential significance of its expression in other carcinomas. This study has several limitations, including the small cohort size, absence of independent positive and negative controls for molecular and immunohistochemical assays, and lack of functional validation experiments. As this pilot study involved a small cohort, the statistical findings are exploratory and hypothesis‐generating, requiring validation in larger populations. Additionally, IHC for EGFRvIII could not be performed due to limited availability of archived tissue samples; future studies using tissue microarrays are planned to validate and complement our molecular findings. Additionally, while IHC represents a useful adjunctive approach, its lower specificity and limited reproducibility compared with molecular techniques should be considered as a methodological limitation. Future studies employing tissue microarrays are warranted to strengthen validation and consistency of the findings. Although our results are consistent with studies in other malignancies where EGFRvIII promotes tumor proliferation, aggressiveness, and treatment resistance [34, 35], the biological relevance of EGFRvIII in gastric carcinoma remains to be confirmed. Future studies should incorporate functional analyses such as cell‐based proliferation or apoptosis assays, drug response profiling, and xenograft models, to elucidate the mechanistic role of EGFRvIII and assess its potential as a therapeutic target.

## 5. Conclusion

Our study revealed that 9.5% of GA samples exhibited elevated levels of EGFRvIII expression. Although the sample size presents certain limitations, an increase in EGFRvIII variant expression was not influenced by the gender or age of the patients. Notably, the association of this variant with the SIG adenocarcinoma subtype is a significant finding that warrants further investigation.

## Ethics Statement

The research was executed after obtaining the approval of the Research Ethics Committee of Iran University of Medical Sciences (IR.IUMS.FMD.REC.1399.241). All patients’ information was kept entirely confidential, and the data analysis was performed without revealing any personal identities of the subjects.

## Disclosure

All authors read and approved the final manuscript.

## Conflicts of Interest

The authors declare no conflicts of interest.

## Author Contributions

Ali Zare‐Mirzaie contributed to the conception and overall design of the study, supervised the pathological evaluation of gastric specimens, critically revised the manuscript, and approved the final version. Narjes Safari participated in the histopathological assessment, data acquisition, and interpretation of clinicopathological findings. Amirhosein Mehrtash participated in the analysis and interpretation of the molecular data. Nasrin Shayanfar assisted in the pathological review of cases, verification of diagnostic criteria, and provided critical comments on the pathology‐related sections of the manuscript. Ensieh Jafari performed the experiments and assisted in data organization. Elmira Gheytanchi performed data analysis and interpretation, drafted the initial version of the manuscript, and integrated co‐authors’ comments into the final version. She acted as the corresponding author and approved the final manuscript on behalf of all authors.

## Funding

This study was supported by Iran University of Medical Sciences.

## Data Availability

The datasets generated and/or analyzed during the current study are available from the corresponding author on reasonable request.
